# Investigating the interplay of stressors and health in horses through fecal cortisol metabolite analysis

**DOI:** 10.3389/fvets.2025.1545577

**Published:** 2025-04-08

**Authors:** Aurelia C. Nowak, Sabine Macho-Maschler, Nora M. Biermann, Rupert Palme, Franziska Dengler

**Affiliations:** ^1^Department of Biological Sciences and Pathobiology, Institute of Physiology and Pathophysiology, University of Veterinary Medicine Vienna, Vienna, Austria; ^2^Department of Biological Sciences and Pathobiology, Experimental Endocrinology, University of Veterinary Medicine Vienna, Vienna, Austria; ^3^Department of Small Animals and Horses, Clinical Unit of Equine Surgery, University of Veterinary Medicine Vienna, Vienna, Austria; ^4^Department of Livestock Tissue Metabolism, Institute of Animal Science, University of Hohenheim, Stuttgart, Germany

**Keywords:** horse/equine, heat, stress, glucocorticoids, disease, pain

## Abstract

**Introduction:**

Horses are highly sensitive to stress, which can affect their wellbeing and lead to various health issues. Effective and objective stress assessment is therefore crucial for improving their care and management. The production of the glucocorticoid cortisol is increased in response to stressful stimuli and its metabolites can be measured non-invasively in feces. Therefore, this study aimed to explore the impact of different environmental and physiological stressors on fecal cortisol metabolite (FCM) concentrations in horses, with a particular focus on the relationship between stress, health, and welfare. We hypothesized that increased FCM levels may be indicative of disease and thus improve early detection and subsequent intervention.

**Methods:**

Fresh fecal samples of *N* = 41 horses (20 geldings and 21 mares) from the same herd were collected once weekly for 1 year. Horses had been housed in the same stable for at least a month and were accustomed to the habitat, daily routine, and social groups. Environmental conditions, health data, and potentially stressful events were recorded. Fecal concentrations of 11,17-dioxoandrostanes were measured via 11-oxoetiocholanolone enzyme immunoassay.

**Results:**

We showed stable baseline FCM concentrations of 6.3 ng/g feces (range 0.6–28.1 ng/g feces). During the summer months, median FCM concentrations increased significantly (*p* < 0.05; One Way RM ANOVA), and this increase correlated with higher ambient temperatures (*p* < 0.0001, *adjr*^2^ = 0.669, Pearson Product Moment correlation). Additionally, other factors such as breed, coat color, and housing conditions influenced the FCM concentrations. Stressful events, such as riding exams and some painful conditions, also resulted in elevated FCM levels, although the magnitude of these responses varied across individual horses. However, not all diseases were associated with increased FCMs.

**Discussion:**

Our findings emphasize the complexity of the hypothalamic-pituitary-adrenal axis in horses, suggesting that while high FCM levels can indicate stress, they may not be reliable biomarkers for early disease detection. Particularly in the light of climate change, the impact of heat stress in the summer months should not be neglected and measures to improve the housing conditions accordingly should become an essential part of equine health management.

## 1 Introduction

Horses are known for a high sympathetic tone and an associated high susceptibility to diseases such as colic, gastric ulcer and generally impaired welfare (1–4). Many stimuli induce increased stress levels in horses, including environmental changes, social interactions, and physical discomfort (5). The activation of the hypothalamic-pituitary axis (HPA) is a key mechanism in regulating this response, aiming to restore homeostasis during episodes of stress or disease (6). In horses, like in many other mammals, the glucocorticoid cortisol is a central mediator in and marker of stress response (7). Although this is widely accepted, it is difficult to objectively measure and quantify stress levels in horses and to establish a causal link to a specific stressor. Understanding the connection between external stimuli and the associated endocrine changes is essential for the promotion of animal welfare (8), which until now often relies on subjective interpretations of animal behavior.

Many studies have measured HPA responses in order to quantify the perceived stress in unfamiliar environments that supposedly impact horses' wellbeing, such as clinical settings, transportation or equestrian events (9–12), and numerous studies have focused on how their bodies respond to painful events (13–15). However, these studies often lack an appropriate control group consisting of animals that experience no disruptions to their daily routine or wellbeing. Furthermore, there are many confounding factors that make it difficult to draw clear conclusions from most of these studies. Assessing stress in animals via plasma, saliva or fecal cortisol (metabolite) concentrations is susceptible to misinterpretation in these contexts, as these attempts to quantify or compare stress responses often fail to account for crucial HPA physiological factors that influence interpretation (16). Many studies were conducted using patients in equine clinics, which means that the horses were transported shortly before sampling, were taken away from their usual environment and social groups to the clinic, where they were subjected to unfamiliar treatments (15, 17). Similar factors play a role in horses sampled during equestrian events. All of these factors, not to mention the original cause for the horses' presence at the clinic, i.e., any kind of potentially painful disease, are supposed to elicit a stress response (17, 18) and therefore may interact and complicate interpretation. Furthermore, most studies performed the measurement only once or twice within a short time frame before exposure to the potential trigger (11, 12, 14), making it difficult to find a valid individual reference value. To ensure more accurate conclusions, it is vital to include matched control groups and establish a solid baseline for comparison, ideally longitudinally for the same horse. Also, a large sample size with consistent conditions would be preferable to accurately identify the external stimuli that truly impact stress responses.

To address these challenges, we measured fecal cortisol metabolite (FCM) concentrations as a non-invasive and reliable method for assessing stress levels in animals (19) longitudinally over a 1 year period in 41 horses of one herd. Blood/plasma cortisol measurements are often utilized as a method to quantify stress response in animals (20–22). However, the sampling procedure itself and the accompanying manipulation of the animal can potentially bias the results (23, 24). Since FCM measurements are non-invasive, less sensitive to fluctuations and the material is easier to collect than other methods such as salivary, urinary or milk glucocorticoid measurements (23) it was our method of choice for this long-term study. A key focus of our research was to examine how FCMs vary in the herd throughout the year and identify factors contributing to these changes with the goal to enhance the understanding of the relationship between stress, environmental factors, and equine health management. Therefore, the aim of the study was to explore the interplay between the horses' health, external influences, and FCM values. Besides establishing a comprehensive overview with our longitudinal study, we hypothesized that the non-invasive measurement of FCMs can support the early detection of diseases in horses.

## 2 Materials and methods

### 2.1 Animals

From November 2022 until the end of October 2023, 41 horses (20 geldings and 21 mares) from a single riding stable in Lower Austria were enrolled in the study. The group contained different breeds: eight Haflinger, six other pony breeds (referred as “Ponies” in the following, consisting of two Hucul ponies, one Fjord horse and three Icelandic horses), three Nordic horses, 18 warm-blooded horses and six Western horses of which five were Quarter horses and one Criollo. Their age ranged from 9 to 27 (15 ± 4.8) years. The stable was located in a rural area at the edge of a large forest area in a continental Pannonian climate. Throughout the whole year, ambient temperatures varied with the seasons, ranging from −9.4°C in winter to 36.3°C in summer. All horses were kept separately in 3 × 4 m boxes with access to 3 × 8 m individual paddocks and had been housed in the stable for at least 1 month prior to study begin. Therefore, they were accustomed to the habitat, daily routine, and social groups. They had free access to water and were fed a standard grain ratio for riding horses consisting of oats and muesli as well as hay three times daily. The horses worked for 45–90 min in riding lessons (mostly dressage or cavaletti training) on a daily basis except weekends between September and June. Riding lessons took place in groups of 4–8 horses being ridden by students (12–18 years old). Due to the consistent training and the animals' body condition scores of 5 ± 1 (assessed by veterinarians twice a year) the fitness of the horses was rated as equal. Additionally, the animals were turned out in group paddocks/pastures once a day for a couple of hours. In the summer months (July–September), the routine of the horses changed, as they were kept in the group paddocks overnight and spent the afternoons in the stables; during these months, the horses were hardly trained. The horses were monitored constantly, and extraordinary events and health data of the horses were recorded by the stable personnel and attending veterinarians.

### 2.2 Fecal cortisol metabolites (FCMs)

Fecal samples were collected from freshly shed feces once weekly (mid-week) at 8 a.m. over a 1-year period and immediately stored at −80°C until further analysis. The sampling was authorized by the ethic commission of the Vetmeduni Vienna (reference number: ETK-030/02/2023).

A total of 2,091 samples (51 samples each from a total of 41 horses) were extracted as described previously (9). In brief, 0.5 g of feces were mixed with 5 mL 80% methanol, vortexed for 30 min and then centrifuged (2,500 × g; 15 min). A volume of 1 ml of the supernatant was transferred into new vials and 5 ml diethylether as well as 0.25 mL 5% NaHCO_3_ added, vortexed for 10 s, centrifuged (2,500 × g; 15 min; 4°C) and then frozen at −20°C overnight. Subsequently, the supernatant was transferred into new vials and dried down before being redissolved in 0.5 mL assay buffer. Concentrations of 11,17-dioxoandrostanes were measured via an 11-oxoetiocholanolone enzyme immunoassay (EIA), which has been described in detail before (25). This EIA has been successfully validated and applied for horses (26, 27).

### 2.3 Statistics

Statistical analysis was performed with Sigma Plot 14.5 (Systat Software Inc., Germany). Data were tested for normality and equal variance using a Shapiro-Wilk test and a Brown-Forsythe test, respectively. One- or two-way repeated measurements (RM) ANOVA was used to identify differences between more than two groups or time points as well as interactions between different factors. Correlation analysis was performed using a Pearson Product Moment correlation. To determine baseline FCM values over a whole year, outliers were identified using GraphPad Prism 10.3.0 (Graphpad Software Inc., USA) and the median of the remaining values was calculated. Graphs were made using either GraphPad Prism or Sigma Plot. Weather data were obtained retrospectively from Meteostat Tulln.

## 3 Results

### 3.1 FCM concentrations over the course of the year

**Figure 1:**
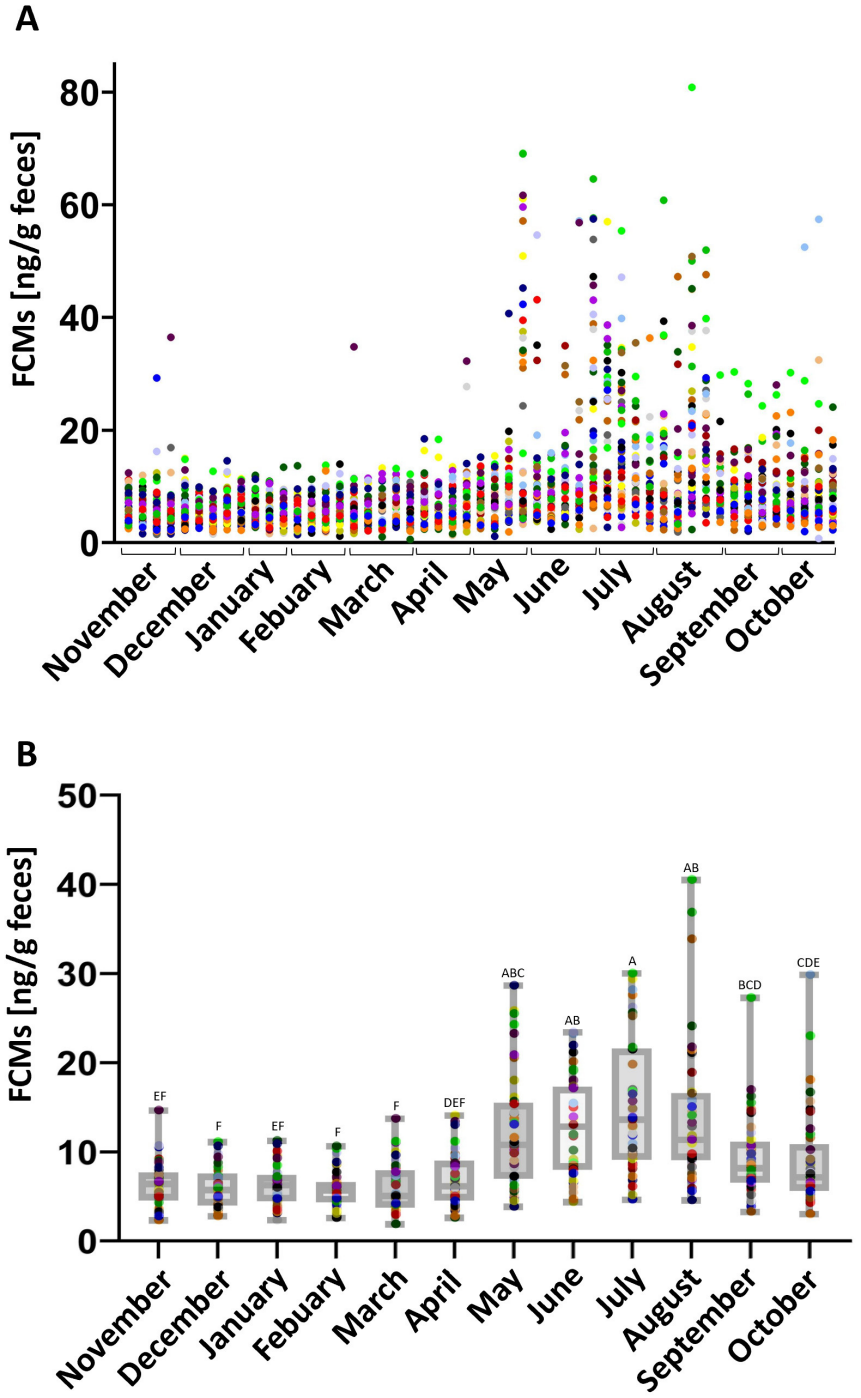
**(A)** Individual FCM values for all horses at all time points sampled. Dots of the same color represent FCM values of one horse for each week over the period of 1 year. **(B)** Median FCM values of all horses for each month. Boxplots show median (line within the box) ± interquartile range (shown by the box) and minimum and maximum values (whiskers), dots represent the median FCM concentrations for each horse in the respective month. Different letters indicate a statistically significant difference between the months (*N* = 41, One Way RM ANOVA, *p* < 0.05).

FCM concentrations were determined once weekly for each horse. A stable baseline of median FCM concentrations of 6.3 (ranging from 0.6 to 28.1) ng/g feces was found. Except for few outliers, all values were almost constant in all horses in the months from September to May. In the summer months, from the end of May until the beginning of September, FCM concentrations increased significantly compared to the rest of the year (*p* < 0.05) and a higher variation between individuals was observed (Figure 1).

### 3.2 Individual characteristics influencing FCM levels

**Figure 2:**
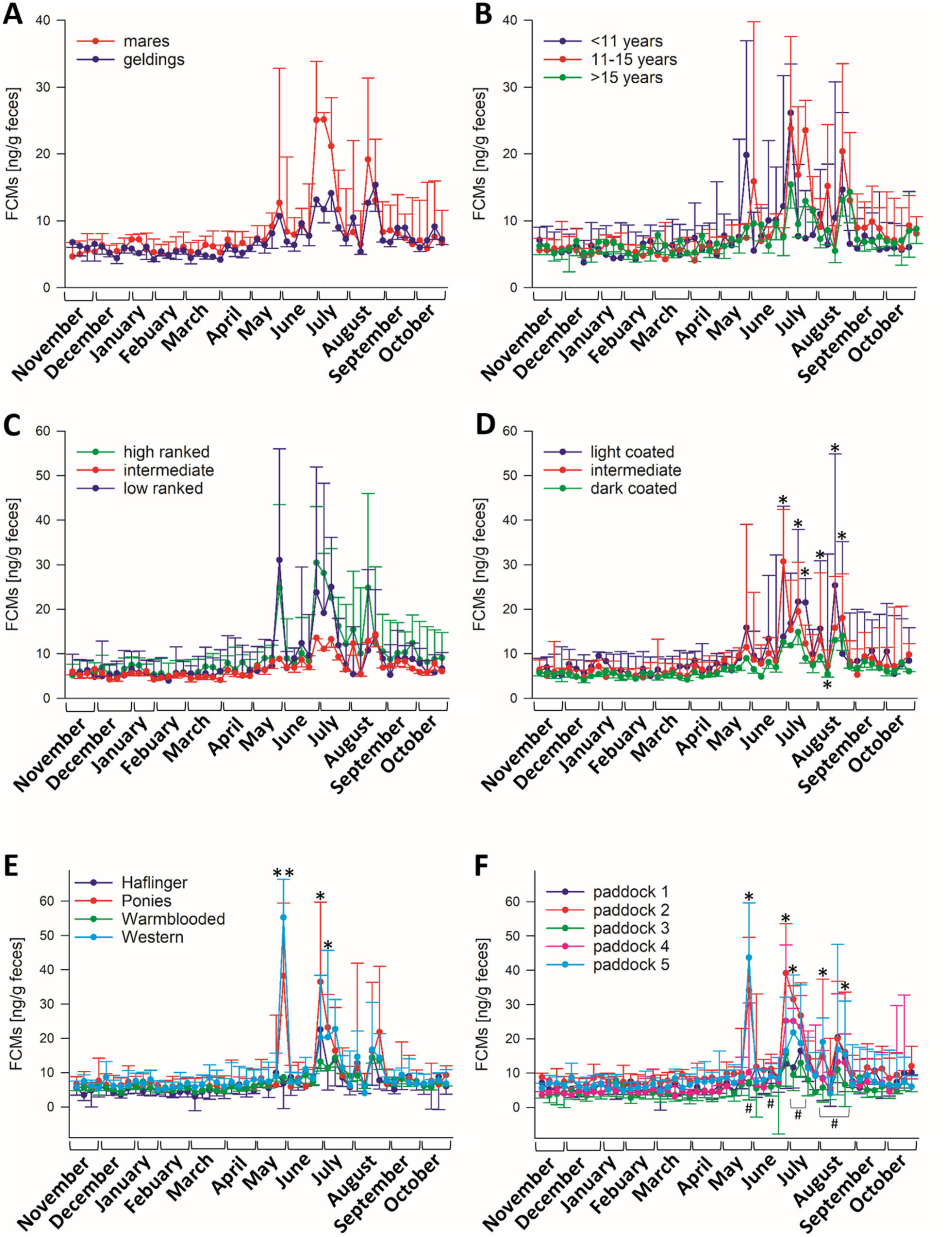
Median (±95% CI) FCM concentrations of horses divided into groups comparing **(A)** sex (*N* = 20 geldings, *N* = 21 mares), **(B)** age (*N* = 8 < 10 years, *N* = 19 11–15 years, *N* = 14 > 16 years), **(C)** rank (*N* = 10 high ranked, *N* = 26 middle, *N* = 5 low ranked horses), **(D)** coat color (*N* = 7 light, *N* = 12 intermediate, *N* = 23 dark coated horses) and **(E)** breed (*N* = 8 Haflingers, *N* = 6 Ponies, *N* = 18 warm-blooded horses, *N* = 6 Western horses), **(F)** paddock groups (paddock 1: *N* = 7, paddock 2: *N* = 8, paddock 3: *N* = 6, paddock 4: *N* = 7, paddock 5: *N* = 8). There were significant interactions between sampling timepoints and groups regarding paddock groups, coat color and breed (*p* < 0.05; Two-Way RM ANOVA), but not for any of the other factors. Asterisks indicate significantly higher values in paddock groups 2 and 5, rhombs indicate significantly lower values in paddock group 3 in comparison to other groups.

To identify factors that might have caused the variation of FCM levels during the summer months, the horses were grouped according to their sex, age, rank in the herd, coat color, breed, and paddock group to test for statistical differences and interactions between the different factors and the sampling time point (Figure 2). There were neither significant differences between male and female horses (Figure 2A), nor regarding the age of the horses (Figure 2B) nor their rank (Figure 2C). None of these factors interacted with the sampling time point. Comparing the coat color (Figure 2D), there was a significant interaction between sampling time point and color (*p* < 0.05) and particularly at those time points when a high variation was observed between the animals (weeks 34, 36, 37, 39, 40, 41, and 42 of sample taking, i.e., end of June to end of August), light coated animals showed significantly higher FCM values than the ones with dark fur, as well as in comparison to intermediate colored horses in weeks 37, 40, and 41 (*p* < 0.05). Also, intermediate-colored horses had significantly higher FCM levels than dark ones in week 34 (*p* < 0.001). Last, we also tested for the influence of the breed, considering the four most frequent breeds in the herd. There was a significant interaction between sampling time point and breed (*p* < 0.001; Figure 2E), with higher FCM concentrations in samples of Western horses and Ponies in comparison to warm-blooded horses and Haflingers in week 29 (*p* < 0.001), while Ponies had higher values in comparison to all other breeds in week 34 (*p* < 0.05) and Western horses had higher concentrations than warm-blooded horses in week 35 (*p* < 0.05). The paddock groups significantly interacted with the sampling timepoints (*p* < 0.05, Figure 2F), and the group comparison revealed results resembling those observed in the breed comparison, as the group composition was quite similar. In weeks 29 and 35, paddocks 2 (primarily Ponies) and 5 (mainly Western horses) had significantly higher FCM values (*p* < 0.05) compared to the other paddock groups. Additionally, paddock 2 showed significantly higher values in weeks 34, 39, and 42 (*p* < 0.05) relative to the other groups. Notably, paddock group 3 displayed significantly lower FCM levels than other groups during weeks 29, 32, 35, 36, 39, 40, 41, and 42 (*p* < 0.05).

### 3.3 FCM levels in correlation with ambient temperature

**Figure 3:**
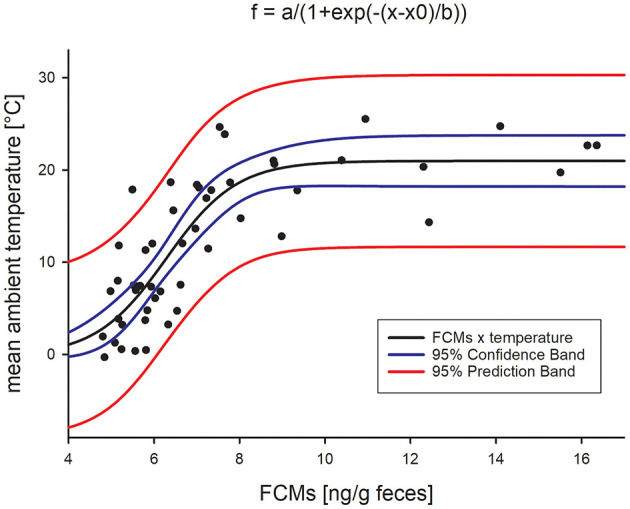
Sigmoidal correlation between mean ambient temperature and median FCM concentrations (Pearson Product Moment Correlation, *adjr*^2^= 0.669, *p* < 0.0001).

As the highest variation of median FCM levels from the baseline appeared to happen particularly in the summer months, we further tested their correlation with ambient temperatures. We found a highly significant sigmoidal correlation with the average ambient temperature per week (Figure 3; *adjr*^2^= 0.669, *p* < 0.0001, Pearson Product Moment Correlation), i.e., with increasing ambient temperatures, FCM levels also increased. However, while the mean temperature did not rise above 25°C, FCM levels were still increasing, thus forming a plateau.

### 3.4 Influence of exams and disease on FCM levels

To identify potential stressors, we measured FCM levels of horses 24 h after a riding exam and compared them with a sample collected 1 week before this event. This time corresponds to the delay time of FCM excretion (28). Another group of horses, which did not participate in the exam, served as a control group. Nonparticipants had median FCM concentrations of 7.6 with a range spanning from a minimum of 2.4 to a maximum of 16.1 ng/g feces 1 week before and 9.2 ranging from a minimum of 4.4 to a maximum of 29.9 ng/g feces on the day after the exam, while participants had values of 7.0 ranging from a minimum of 3.9 to a maximum of 13.3 ng/g feces 1 week before and 11.6 with minimum values of 3.2 and maximum values of 69.2 ng/g feces 1 day after the exam.

**Figure 4:**
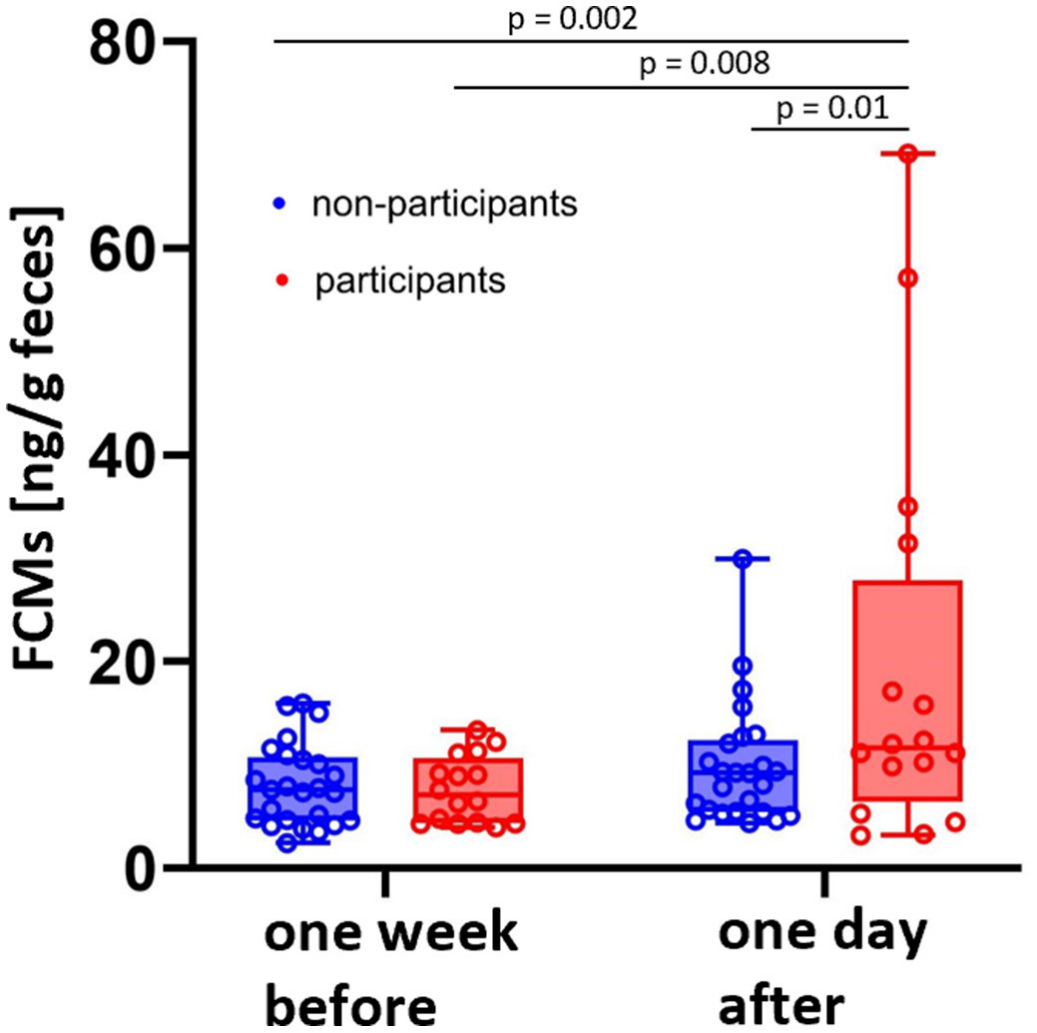
Median FCM concentrations of horses participating in a riding exam were higher than baseline values measured 1 week before (*N* = 16; red boxes) as well as compared with non-participating herd mates (*N* = 25; blue boxes) (One-Way ANOVA). Boxplots show median (line within the box) ± interquartile range (shown by the box) and minimum and maximum values (whiskers). Dots represent the FCM concentrations for each horse at the respective timepoint.

Both, the comparison between the groups as well as between timepoints, resulted in significantly higher FCM concentrations in the group of participating animals 24 h after the exam compared to the control group as well as the values of the same horses 1 week before the exam (*p* < 0.05; Figure 4).

**Figure 5:**
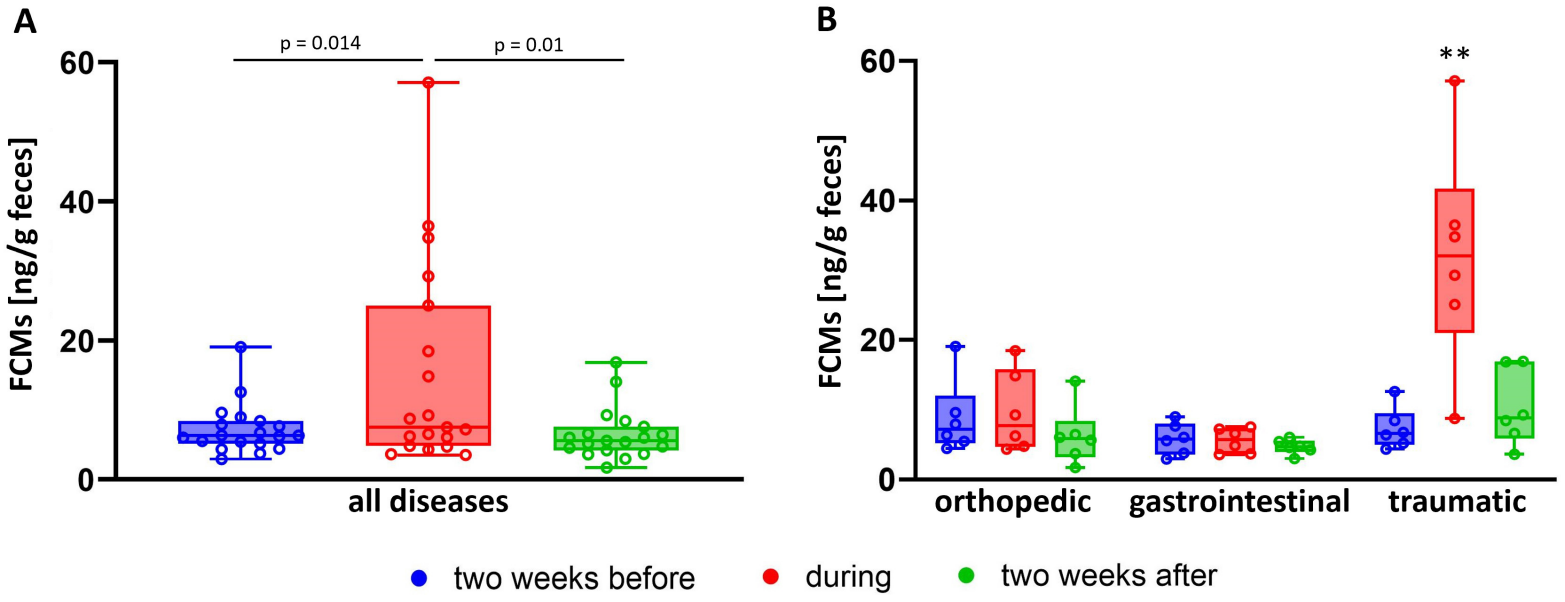
Comparison of FCM values in **(A)** diseased horses (*N* = 18) before (blue boxes), during (red boxes), and after (green boxes) the reported disease resulting in significantly increased values during the time of disease (One Way RM ANOVA), as well as **(B)** distinguishing the etiology of the disease showing that traumatic injuries (*N* = 6) significantly increased FCM concentrations, whereas orthopedic (*N* = 6) and gastrointestinal disease (*N* = 6) did not (Two Way RM ANOVA; **p < 0.01). Boxplots show median (line within the box) ± interquartile range (shown by the box) and minimum and maximum values (whiskers), dots represent the median FCM concentrations for each horse at the respective timepoint.

Besides excitement due to exams, disease is supposed to be another stressful stimulus in horses (9). We compiled FCM values of horses, that were examined by a veterinarian for various symptoms during the acute diseased phase and compared these to the values of the same horses 2 weeks before and after. FCM concentrations were increased during the acute phase of disease (median = 8.1; minimum = 3.6; maximum = 57.1 ng/g feces) compared to the values before (median = 6.4; minimum = 2.9; maximum = 19.1 ng/g feces) and after (median = 5.8; minimum = 1.7; maximum = 16.9 ng/g feces) (One Way RM ANOVA; *p* < 0.05; Figure 5A). However, FCM values appeared not to be increased in all horses during all pathological events. Hence, we further subdivided the diseases in orthopedic lesions (mainly lameness), gastrointestinal disease (diarrhea or fecal water) and traumatic, potentially painful diseases (injuries, edema, swellings) (Figure 5B). By comparing these groups, we could confirm increased FCM levels during the acute phase of the disease in all groups (*p* < 0.01, Two Way RM ANOVA), however this appeared to be mainly due to the group of horses with traumatic diseases which had significantly higher FCM concentrations not only during the acute phase of the disease compared to other stages, but also in comparison to groups with different etiologies at any timepoint (Two Way RM ANOVA; *p* < 0.001)

## 4 Discussion

This study was conducted to measure FCM concentrations in a large group of horses over the course of a whole year and aimed to identify reliable baseline values for FCM levels in horses as well as factors leading to increased FCM levels. We hypothesized that disease processes lead to elevated FCM levels, which might thus serve as early indicator for the need of medical intervention. The findings of the present study revealed a steady baseline of median FCM concentrations in the study population during the months from September until May, which corresponds to the physiological values (0.7–10.7 ng/g feces) described earlier in a smaller group of six mares over a time period of 6 days (26). Our findings are consistent with previous research using salivary and plasma cortisol measurements. Several studies demonstrated that plasma cortisol concentrations correlate with both salivary cortisol levels (21, 29) as well as with FCM values (27, 30). For instance, Pawluski et al. reported evening plasma cortisol concentrations of 12.6 ± 1.1 ng/mL in a group of 59 horses after a rest day, with corresponding FCM levels of 4.9 ± 0.4 ng/g feces (30). Another study including 55 horses found plasma cortisol concentrations ranging from 2.5 to 40.3 ng/mL while FCMs ranged from 1.6 to 13.1 ng/g feces (27). These results align closely with the baseline values observed in our study. Particularly in the summer months the FCM concentrations were increased and showed greater variability between individuals. However, we did not observe an increase in FCM values across all horses, which may be attributed to different stress levels in individual horses or individual variations in the stress response.

One explanation for increased values in the summer months might be sex differences, anticipating that mares undergo hormonal changes associated with their reproductive cycles during this time of the year, as horses are long-day breeders (31, 32). However, our analysis revealed no significant differences in overall values between mares and geldings. Still, greater variation of the FCM values and a clearly visible peak in early summer in mares compared to geldings suggest that sex may have some effect on FCM values. Since former life experience (33) as well as the social status of the animal (34) can have an impact on individual FCM concentrations, factors like age and rank order should also not be neglected, although we did not observe an impact of these factors on FCM levels. This could be due to a pre-existing hierarchy within the herd resulting in less severe aggressive interactions (35). However, there was an interaction between FCM values and sampling timepoint in relation to the paddock groups. Nevertheless, it is unlikely that this interaction was driven by social stress, as the horses were kept in consistent, fixed groups in the paddocks throughout the year.

While social factors appeared to have no influence on FCM concentrations, our investigations revealed a highly significant correlation between FCM values and the ambient temperature, suggesting an elevated stress response due to rising temperatures in the summer months. Even though there is no clear definition of heat stress in horses (36) rising ambient temperatures activate the HPA in mammals, resulting in the release of glucocorticoids (37). In addition to their impact on immunity, metabolism, neurobiology, and reproductive physiology, glucocorticoids also mediate cardiovascular activation increasing blood pressure and cardiac output as well as promoting vasodilation to facilitate heat dissipation within the body's thermoregulation system (38). In the weeks 29, 34, and 35 of the sampling period ambient temperatures rose up to 33°C, overstepping the thermoneutral zone of horses ranging from 5 to 25°C (39). Accordingly, median FCM values reached the highest levels at these time points. Different horse breeds have distinct heat tolerance according to the adaptation to their original environment (40). This is also reflected in our data showing a significant interaction between the breed and the sampling timepoint. During the summer months, the Western horses and the Ponies had a stronger stress response mirrored by higher FCM concentrations in warmer conditions. Although the group of Ponies was rather heterogenous, they all share an origin from Northern regions, where the weather conditions are different to those experienced in central Europe. Icelandic horses have originally been bred to live in the rough climate of Iceland and the same applies for Hucul ponies, bred primarily in the Carpathian Mountains and Fjord horses, which have their origin in Norway, whereas the origin of other robust pony breeds such as Haflingers lies in warmer regions such as Tirol and South Tirol. Morphological features, such as thicker coats, help minimize heat loss from the animal (41). Additionally, small breeds have a higher surface area to body weight relation compared to warm-blooded horses, making them more susceptible to solar radiation (42). Other phenotypical factors such as the length of the legs also play a crucial role, since animals with long, slender limbs experience less heat absorption and greater heat dissipation (43). All of these factors may have a role in the thermoregulatory capacities and lead to an increased stress response to heat of the Ponies in this study. Western horses (represented in this study by five Quarter horses and one Criollo) also had a higher stress response when temperatures rose. This may be attributed to variations in the thermoneutral zone. For example, winter-acclimatized adult Quarter horse geldings exhibited their lowest metabolic rate at temperatures ranging from −10 to +10°C (44). These findings are further supported by the significantly higher FCM values observed in paddock groups 2, predominantly consisting of Ponies, and 5, which primarily housed Western horses. In addition to the temperature, relative humidity is a major factor determining heat stress. Unfortunately, there was no data available for humidity in the area during the experimental phase and could thus not be taken into account.

Additionally, we found an interaction between coat color and sampling timepoint. In combination with the correlation between ambient temperatures and FCM levels, this may indicate varying levels of heat tolerance based on coat color. It is widely accepted that black-coated animals absorb more heat than their white counterparts (45), which would suggest that they experience greater heat stress. However, in our study light-coated horses had higher FCM levels. These results align with those of Al-Haidary et al. (46), who reported that sheep with black coats exhibited better heat tolerance compared to white coated ones, evidenced by lower rectal temperatures and higher heat tolerance indices. Multiple studies examining the measurement of horses' body temperature in different setting showed increased body temperatures in warmer ambient conditions (47–49), although no differences related to coat color were observed (47, 48). Furthermore, hair characteristics have a greater influence on animal performance under thermal stress than coat color (50). Considering that Nordic pony breeds have thicker coats, both characteristics appear to play a role in our study. Interestingly, one light coated horse had particularly high FCM levels during the summer months without any obvious explanation and might therefore have (over-)emphasized the high values in this group. This can be considered as an indicator that also the horses' individual temperament influences their susceptibility to various triggers. The higher FCM values in light coated horses might also indicate that insects prefer light surfaces, and thus light-colored horses were bothered more than others. In addition to the heat, the summer months imply an increase in biting and bothering insects, which can cause irritation and stress for the horses (51). According to the subjective perception of the stable staff, insect infestation in the stable was clearly noticeable. Insect repellent sprays were used only rarely and only on some horses and no additional fly protection such as masks or blankets was used. This means that most of the animals in this study were exposed to biting insects, especially in the paddocks. These factors may also trigger a heightened stress response, which could explain why FCM concentrations often remained high or increased even more, although ambient temperatures did not increase further, rather than correlating in a linear manner with temperature changes.

Another explanation for the increased FCM values could be that during the summer months the horses were kept in the paddocks overnight. This assumption is in accordance with a study showing significantly higher hair cortisol concentrations in horses that were kept in paddocks overnight compared to horses from the same stable kept indoors (52). Spending the night on the paddock could interfere with the sleep quality of the animals, especially when considering that horses are flight animals. Horses bedded on straw spent more hours in recumbency than individuals with other beddings (53). In our study the horses were kept in straw bedded boxes, but when left in the paddocks overnight during summer, no bedding was provided, although they had access to shelters with two closed sides and a roof. This, in combination with contact to wildlife, which may have occurred during the night, could be a contributing factor to elevated FCM levels in these months. This notion is supported by the observation that FCM levels returned to the observed baseline values almost immediately after resuming the daily routine of keeping the horses in the stable at night and turning them out in the afternoon in September. Although the sleep routine of the animals was not assessed in this study, the comparison of the paddock groups showed that horses turned out in the paddock closest to the stable and farthest from the woods had significantly lower FCM values than those in other groups, further underlining the prior assumption. In contrast, the paddock for the group including mostly the Ponies is located closest to the edge of the adjoining forest. Interestingly, one of the Icelandic horses that was turned out on another, smaller paddock closer to the stable, showed stable FCM values throughout the whole year and did not contribute to the overall significantly higher FCM levels in this group compared to other breeds. These findings suggest that the paddock location, and thus potential contact with wildlife, may have a greater impact on the animals' stress response than breed. However, when considering the paddock placement of the Western horses, we observed that two warm-blooded horses housed in the same paddock together with five out of six Western horses had stable and lower FCM levels compared to the Western horses. This suggests that in this case breed may have a stronger influence. Given the combination of these two factors (breed and potential contact with wildlife), and the small number of animals in each subgroup, particularly for Ponies and Western horses, it is difficult to establish a clear causal link between either factor and the higher FCM levels in our study. It is also important to note that all animals in this study were from the same herd, which introduces the potential for pseudoreplication. Future studies should aim to include horses from several herds to mitigate this issue and strengthen the robustness of the findings.

Our data also unveiled other stressors triggering the activation of the HPA. A riding exam, which took place at the same site where the horses were housed, appeared to have had an impact on the stress response of the animals, since FCM values increased 24 h after this event. Although FCM levels did not increase in all of the participating horses, the median FCM values before and after the event differed significantly. One reason to explain this could be the different levels of experience and riding ability of the participating equestrians. Wolframm and Micklewright (54) highlighted the significance of varying levels of pre-competitive arousal, revealing that riders who interpret their arousal in a more positive way tend to perform better. Given that the relationship between horse and rider relies heavily on non-verbal communication, any signs of anxiety from the rider could be perceived as a threat by the horse, potentially triggering a flight response. It was also demonstrated that elite equestrians, in contrast to non-elite riders, experience lower levels of somatic arousal and higher levels of self-confidence, which allows them to communicate more effectively with their horses (55). To better assess this topic, further research considering the riders' stress level during the event is warranted. Furthermore, it must be noted that the regulation of the HPA is complex and increased FCM levels may not have one single cause.

Pathological events and pain trigger the activation of the HPA to help restore the body's homeostasis (6). Therefore, increased FCM levels may be indicative of disease. A study that examined plasma cortisol levels in horses over a 24-h period revealed that cortisol levels in horses undergoing emergency surgery for acute gastrointestinal disease significantly increased (10). Merl et al. (9) measured FCM concentrations in stallions before and after castration and found significantly higher values 2 days after the procedure. This suggests that painful experiences result in elevated FCM concentrations in horses. Our investigations revealed that the FCM values in horses at the time of disease were significantly increased compared to those recorded 2 weeks before or after. However, not all horses had elevated FCM concentrations during this period. This variation may be attributed to the severity of the disease and the individual horse's perception of pain. When categorizing diseases based on their etiology, it was obvious that painful events such as traumatic injuries had a greater impact on FCM levels than orthopedic or gastrointestinal conditions. As also shown in other studies (10, 17), this may be attributed to the intensity of pain experienced by the horse. Assessing a pain scale or the heart rate (variability) of the horses could have further supported this interpretation but was unfortunately not feasible in our study. These findings suggest that while some painful events may be reflected in elevated FCM values, FCMs are not a consistently reliable indicator for disease detection in general.

## 5 Conclusion

In conclusion, our study demonstrates that FCM concentrations correlated significantly with ambient temperatures, indicating that heat stress in horses was reflected in these measurements. Specifically, Ponies, Western horses, and light-coated horses appeared to be more susceptible to heat stress, as evidenced by elevated FCM levels. Additionally, factors such as insect infestations and changes in housing conditions—particularly alterations in the animals' sleeping routines—may impact FCM values, further highlighting the complexity of HPA responses in horses. Stressful events, such as riding exams, also increased FCM levels. While some pathological processes influenced FCM values, their impact varied based on the severity of the condition, making FCMs a less reliable indicator for prediction of diseases.

## Data Availability

The raw data supporting the conclusions of this article are available from the corresponding authors upon reasonable request.

## References

[1] Scheidegger MD Gerber V Bruckmaier RM van der Kolk JH Burger D Ramseyer A. Increased adrenocortical response to adrenocorticotropic hormone (ACTH) in sport horses with equine glandular gastric disease (EGGD). Vet J. (2017) 228:7–12. 10.1016/j.tvjl.2017.09.002 29153110

[2] Sykes BW Hewetson M Hepburn RJ Luthersson N Tamzali Y. European college of equine internal medicine consensus statement—equine gastric ulcer syndrome in adult horses. J Vet Intern Med. (2015) 29:1288–99. 10.1111/jvim.13578 26340142 PMC4858038

[3] Leal BB Alves GE Douglas RH Bringel B Young RJ Haddad JPA. Cortisol circadian rhythm ratio: a simple method to detect stressed horses at higher risk of colic? J Equine Vet Sci. (2011) 31:188–90. 10.1016/j.jevs.2011.02.005

[4] Keadle TL Pourciau SS Melrose PA Kammerling SG Horohov DW. Acute exercises stress modulates immune function in unfit horses. J Equine Vet Sci. (1993) 13:226–31. 10.1016/S0737-0806(06)81019-1

[5] Mormède P Andanson S Aupérin B Beerda B Guémené D Malmkvist J et al. Exploration of the hypothalamic–pituitary–adrenal function as a tool to evaluate animal welfare. Physiol Behav. (2007) 92:317–39. 10.1016/j.physbeh.2006.12.003 17234221

[6] Wagner AE. Effects of stress on pain in horses and incorporating pain scales for equine practice. Vet Clin North Am Equine Pract. (2010) 26:481–92. 10.1016/j.cveq.2010.07.001 21056295

[7] Bruijn R de Romero LM. The role of glucocorticoids in the vertebrate response to weather. Gen Comp Endocrinol. (2018) 269:11–32. 10.1016/j.ygcen.2018.07.007 30012539

[8] Etim N Offiong E Evans EI Akpabio U. Endocrine system: indicators of stress and a means of evaluating animal welfare. Eur Int J Sci Technol. (2013) 2:141–8.

[9] Merl S Scherzer S Palme R Möstl E. Pain causes increased concentrations of glucocorticoid metabolites in horse feces. J Equine Vet Sci. (2000) 20:586–90. 10.1016/S0737-0806(00)70267-X

[10] Pritchett LC Ulibarri C Roberts MC Schneider RK Sellon DC. Identification of potential physiological and behavioral indicators of postoperative pain in horses after exploratory celiotomy for colic. Appl Animal Behav Sci. (2003) 80:31–43. 10.1016/S0168-1591(02)00205-8

[11] Olvera-Maneu S Carbajal A Serres-Corral P López-Béjar M. Cortisol variations to estimate the physiological stress response in horses at a traditional equestrian event. Animals. (2023) 13:396. 10.3390/ani13030396 36766285 PMC9913708

[12] Jacquay ET Harris PA Adams AA. Age-related differences in short-term transportation stress responses of horses. J Equine Vet Sci. (2023) 128:104879. 10.1016/j.jevs.2023.104879 37399910

[13] Raekallio M Taylor PM Bennett RC. Preliminary investigations of pain and analgesia assessment in horses administered phenylbutazone or placebo after arthroscopic surgery. Vet Surg. (1997) 26:150–5. 10.1111/j.1532-950X.1997.tb01478.x 9068166

[14] Rowland AL Glass KG Grady ST Cummings KJ Hinrichs K Watts AE. Influence of caudal epidural analgesia on cortisol concentrations and pain-related behavioral responses in mares during and after ovariectomy via colpotomy. Vet Surg. (2018) 47:715–21. 10.1111/vsu.12908 29774961

[15] Gehlen H Faust M-D Grzeskowiak RM Trachsel DS. Association between disease severity, heart rate variability (HRV) and serum cortisol concentrations in horses with acute abdominal pain. Animals. (2020) 10:1563. 10.3390/ani10091563 32887514 PMC7552187

[16] Hart KA. The use of cortisol for the objective assessment of stress in animals: Pros and cons. Vet J. (2012) 192:137–9. 10.1016/j.tvjl.2012.03.016 22534187

[17] Mair TS Sherlock CE Boden LA. Serum cortisol concentrations in horses with colic. V J. (2014) 201:370–7. 10.1016/j.tvjl.2014.06.005 24986316

[18] Schmidt A Möstl E Wehnert C Aurich J Müller J Aurich C. Cortisol release and heart rate variability in horses during road transport. Horm Behav. (2010) 57:209–15. 10.1016/j.yhbeh.2009.11.003 19944105

[19] Palme R. Non-invasive measurement of glucocorticoids: Advances and problems. Physiol Behav. (2019) 199:229–43. 10.1016/j.physbeh.2018.11.021 30468744

[20] Hovey MR Davis A Chen S Godwin P Porr CAS. Evaluating stress in riding horses: part one-behavior assessment and serum cortisol. J Equine Vet Sci. (2021) 96:103297. 10.1016/j.jevs.2020.103297 33349400

[21] Kedzierski W Cywińska A Strzelec K Kowalik S. Changes in salivary and plasma cortisol levels in Purebred Arabian horses during race training session. Anim Sci J. (2014) 85:313–7. 10.1111/asj.12146 24261657

[22] Soroko M Howell K Dudek K Waliczek A Micek P Flaga J. Relationship between maximum eye temperature and plasma cortisol concentration in racehorses during intensive training. Pol J Vet Sci. (2021) 24:393–7. 10.24425/pjvs.2021.138730 34730308

[23] Palme R. Monitoring stress hormone metabolites as a useful, non-invasive tool for welfare assessment in farm animals. Anim Welf. (2012) 21:331–7. 10.7120/09627286.21.3.331

[24] Share ER Mastellar SL Suagee-Bedore JK Eastridge ML. Validation of a commercial ELISA kit for non-invasive measurement of biologically relevant changes in equine cortisol concentrations. Animals. (2024) 14:2831. 10.3390/ani14192831 39409780 PMC11475127

[25] Palme R Möstl E. Measurement of cortisol metabolites in faeces of sheep as a parameter of cortisol concentration in blood. Int J Mammal Biol. (1997) 62:192–7.27167500

[26] Möstl E Messmann S Bagu E Robia C Palme R. Measurement of glucocorticoid metabolite concentrations in faeces of domestic livestock. Zentralbl Veterinarmed A. (1999) 46:621–31. 10.1046/j.1439-0442.1999.00256.x 10638300

[27] Fureix C Benhajali H Henry S Bruchet A Prunier A Ezzaouia M. Plasma cortisol and faecal cortisol metabolites concentrations in stereotypic and non-stereotypic horses: do stereotypic horses cope better with poor environmental conditions? BMC Vet Res. (2013) 9:3. 10.1186/1746-6148-9-3 23289406 PMC3544618

[28] Palme R. Measuring fecal steroids: guidelines for practical application. Ann N Y Acad Sci. (2005) 1046:75–80. 10.1196/annals.1343.007 16055844

[29] Peeters M Sulon J Beckers J-F Ledoux D Vandenheede M. Comparison between blood serum and salivary cortisol concentrations in horses using an adrenocorticotropic hormone challenge. Equine Vet J. (2011) 43:487–93. 10.1111/j.2042-3306.2010.00294.x 21496072

[30] Pawluski J Jego P Henry S Bruchet A Palme R Coste C. Low plasma cortisol and fecal cortisol metabolite measures as indicators of compromised welfare in domestic horses (*Equus caballus*). PLoS ONE. (2017) 12:e0182257. 10.1371/journal.pone.0182257 28886020 PMC5590897

[31] Aurich C. Reproductive cycles of horses. Anim Reprod Sci. (2011) 124:220–8. 10.1016/j.anireprosci.2011.02.005 21377299

[32] Hicks GR Fraser NS Bertin F-R. Changes associated with the peri-ovulatory period, age and pregnancy in ACTH, cortisol, glucose and insulin concentrations in mares. Animals. (2021) 11:891. 10.3390/ani11030891 33804751 PMC8003915

[33] Koolhaas JM Bartolomucci A Buwalda B Boer SF de Flügge G Korte SM. Stress revisited: a critical evaluation of the stress concept. Neurosci Biobehav Rev. (2011) 35:1291–301. 10.1016/j.neubiorev.2011.02.003 21316391

[34] Creel S Dantzer B Goymann W Rubenstein DR. The ecology of stress: effects of the social environment. Funct Ecol. (2013) 27:66–80. 10.1111/j.1365-2435.2012.02029.x

[35] York CA Schulte BA. The relationship of dominance, reproductive state and stress in female horses (*Equus caballus*). Behav Process. (2014) 107:15–21. 10.1016/j.beproc.2014.07.005 25058621

[36] Kang H Zsoldos RR Sole-Guitart A Narayan E Cawdell-Smith AJ Gaughan JB. Heat stress in horses: a literature review. Int J Biometeorol. (2023) 67:957–73. 10.1007/s00484-023-02467-7 37060454 PMC10267279

[37] Gonzalez-Rivas PA Chauhan SS Ha M Fegan N Dunshea FR Warner RD. Effects of heat stress on animal physiology, metabolism, and meat quality: a review. Meat Sci. (2020) 162:108025. 10.1016/j.meatsci.2019.108025 31841730

[38] Sapolsky RM Romero LM Munck AU. How do glucocorticoids influence stress responses? Integrating permissive, suppressive, stimulatory, and preparative actions. Endocr Rev. (2000) 21:55–89. 10.1210/edrv.21.1.0389 10696570

[39] Morgan K. Thermoneutral zone and critical temperatures of horses. J Therm Biol. (1998) 23:59–61. 10.1016/S0306-4565(97)00047-8

[40] Castanheira M Rezende Paiva S Louvandini H Landim A Clorinda Soares Fiorvanti M Regina Paludo G et al. Multivariate analysis for characteristics of heat tolerance in horses in Brazil. Trop Anim Health Prod. (2010) 42:185–91. 10.1007/s11250-009-9404-x 19579053

[41] Leite JH Da Silva RG Asensio LAB de Sousa JER Da Silva WST Da Silva WE et al. Coat color and morphological hair traits influence on the mechanisms related to the heat tolerance in hair sheep. Int J Biometeorol. (2020) 64:2185–94. 10.1007/s00484-020-02014-8 32918600

[42] West JW. Effects of heat-stress on production in dairy cattle. J Dairy Sci. (2003) 86:2131–44. 10.3168/jds.S0022-0302(03)73803-X 12836950

[43] Mwacharo JM Okeyo AM Kamande GK Rege JEO. The small east african shorthorn zebu cows in Kenya. I: linear body measurements. Trop Anim Health Prod. (2006) 38:65–74. 10.1007/s11250-006-4266-y 17405630

[44] McBride GE Christopherson RJ Sauer W. Metabolic rate and plasma thyroid hormone concentrations of mature horses in response to changes in ambient temperature. Can J Anim Sci. (1985) 65:375–82. 10.4141/cjas85-043

[45] Stewart RE. Absorption of solar radiation by the hair of cattle. Agric Engng. (1953) 34:235–8.

[46] Al-Haidary AA Al-Dosari Y Abd-Elwahab A-E Samara EM Al-Badwi MA Abdoun KA. White hair coat color does not influence heat tolerance of sheep grazing under a hot arid environment. Small Rumin Res. (2021) 201:106410. 10.1016/j.smallrumres.2021.106410

[47] Robinson TR Hussey SB Hill AE Heckendorf CC Stricklin JB Traub-Dargatz JL. Comparison of temperature readings from a percutaneous thermal sensing microchip with temperature readings from a digital rectal thermometer in equids. J Am Vet Med Assoc. (2008) 233:613–7. 10.2460/javma.233.4.613 18710319

[48] Verdegaal E-L Howarth GS McWhorter TJ Delesalle CJG. Is continuous monitoring of skin surface temperature a reliable proxy to assess the thermoregulatory response in endurance horses during field exercise? Front Vet Sci. (2022) 9:894146. 10.3389/fvets.2022.894146 35711810 PMC9196037

[49] Brownlow M Smith T. The use of the hand-held infrared thermometer as an early detection tool for exertional heat illness in Thoroughbred racehorses: A study at racetracks in eastern Australia. Equine Vet Educ. (2021) 33:296–305. 10.1111/eve.13299

[50] Farias CO Lazzari J Da Soares Cunha Í Gonçalves PBD Gasperin BG Lucia T et al. Thermotolerance in Angus cattle is related to hair coat characteristics but not to coat color. J Therm Biol. (2024) 124:103945. 10.1016/j.jtherbio.2024.103945 39142266

[51] Genchi M Kramer L Valentini G Allievi G Ciuca L Vismarra A. Efficacy of topical administration of prallethrin-permethrin-piperonyl butoxide (Bronco^®^ Equine Fly Spray) for the treatment and control of flies and other nuisance insects of horses. Parasitol Res. (2023) 122:3139–45. 10.1007/s00436-023-08004-0 37921904 PMC10667147

[52] Mazzola SM Colombani C Pizzamiglio G Cannas S Palestrini C Costa ED. Do you think i am living well? A four-season hair cortisol analysis on leisure horses in different housing and management conditions. Animals. (2021) 11:2141. 10.3390/ani11072141 34359269 PMC8300697

[53] Kwiatkowska-Stenzel A Sowińska J Witkowska D. The effect of different bedding materials used in stable on horses behavior. J Equine Vet Sci. (2016) 42:57–66. 10.1016/j.jevs.2016.03.007

[54] Wolframm IA Micklewright D. Effects of trait anxiety and direction of pre-competitive arousal on performance in the equestrian disciplines of dressage, showjumping and eventing. Comp Exercise Physiol. (2010) 7:185–91. 10.1017/S1755254011000080

[55] Wolframm IA Micklewright D. Pre-competitive levels of arousal and self-confidence among elite and non-elite equestrian riders. Comp Exercise Physiol. (2008) 5:153–9. 10.1017/S1478061509356133

